# Starving cancer cells to enhance DNA damage and immunotherapy response

**DOI:** 10.18632/oncotarget.28595

**Published:** 2024-06-20

**Authors:** Aashirwad Shahi, Dawit Kidane

**Affiliations:** ^1^Department of Physiology and Biophysics, College of Medicine, Howard University, Washington, DC 20059, USA

**Keywords:** amino acid depletion, DNA damage, DNA repair, Immunotherpy, tumor immunity

## Abstract

Prostate cancer (PCa) poses significant challenges in treatment, particularly when it progresses to a metastatic, castrate-resistant state. Conventional therapies, including chemotherapy, radiotherapy, and hormonal treatments, often fail due to toxicities, off-target effects, and acquired resistance. This research perspective defines an alternative therapeutic strategy focusing on the metabolic vulnerabilities of PCa cells, specifically their reliance on non-essential amino acids such as cysteine. Using an engineered enzyme cyst(e)inase to deplete the cysteine/cystine can induce oxidative stress and DNA damage in cancer cells. This depletion elevates reactive oxygen species (ROS) levels, disrupts glutathione synthesis, and enhances DNA damage, leading to cancer cell death. The combinatorial use of cyst(e)inase with agents targeting antioxidant defenses, such as thioredoxins, further amplifies ROS accumulation and cytotoxicity in PCa cells. Overall, in this perspective provides a compressive overview of the previous work on manipulating amino acid metabolism and redox balance modulate the efficacy of DNA repair-targeted and immune checkpoint blockade therapies in prostate cancer.

## INTRODUCTION

Prostate cancer (PCa) is a main cancer types that affects men’s health and poorly responded to treatment once it becomes metastatic [1, 2]. If PCa cells depend on androgen for their growth, it provides successful treatment option for patients with androgen deprivation therapy [3]. In androgen positive PCa, the second-generation anti-androgens drugs such as abiraterone or enzalutamide have provided modest survival benefits of ~4–5 months, [4, 5]. However, the disease can eventually progress to an androgen-independent state known as castrate resistant prostate cancer (CRPC) and becomes unresponsive to chemotherapy, radiotherapy, or hormonal therapy [6–8]. One of the challenges in treating cancer with chemotherapies resulting in toxicities and off target effect toward normal cells at the doses required to kill tumor cells. Furthermore, limited number of PCa harbor actionable genomic aberrations that may respond to targeted therapy [9, 10]. This scenario is occurred due to several factors including the presence of co-occurring genomic alterations, intra-patient tumor heterogeneity, and the development of acquired resistance. Due to those challenges alternative therapeutic strategies are required to improve treatment response or overcome resistance.

Targeting cancer cell metabolism may improve treatment response in therapy resistant cancers and reduces treatment-related toxicities. One of the hallmarks of PCa is metabolic reprogramming and is represented by the dependency of tumors on metabolic pathways for promoting growth, survival [11]. The link between cancer and dysregulated metabolism discovered during the early period of cancer biology and explained on Warburg effect [12]. This article focuses on alterations in the metabolism of PCa, genetic signatures and molecular pathways associated with non- essential amino acids metabolic needs of the cancer cells. It is well known fact that amino acids perform critical metabolic functions. Amino acid-depletion therapies target amino acid uptake and catabolism using heterologous enzymes or recombinant or engineered human enzymes. Notably, such therapies have minimal effect on normal cells due to their lower demand for amino acids compared with tumor cells. Normal cells exhibit lower demand for amino acids [13]. In contrast, cancer cell has high demand of amino acids due to meet increased demands for energy and cellular building blocks that leads to use extracellular pool of amino acids. One of the therapeutic strategies that satisfies the objective of developing cancer cell-selective therapeutics is the systemic depletion of that tumor-essential amino acid, which can result in tumor apoptosis with minimal side effects to normal cells. In recent years, Cramer et al. 2017 developed the engineered cyst(e)inase enzyme that able to degrade intracellular and extracellular cysteine/cystine [14]. Furthermore, Cyst(ei)nase enzyme able to decrease the antioxidant glutathione synthesis. In this scenario, our follow up studies have shown that depletion of amino acids such as cystine and cysteine in different cancer cell types help to design combinatorial therapeutic approaches using DNA repair targeted therapy and immune checkpoint blockade for the attainment of more durable therapeutic responses. Those metabolic vulnerabilities and loss of normal fitness of the cancer cells to amino acid depletion likely provide additional novel therapeutic options in cancer. In this prospective, we will rise the driving questions and potential possibilities how amino acid depletion induced oxidative stress associated DNA damage exploited for DNA repair targeted and immune checkpoint blockade therapy in the next section of this manuscript.

### Driving questions

Exploiting amino acid metabolism can be done via different mechanisms including inhibition of either amino acid transporters [15], amino acid biosynthesis, or by depletion of amino acids. Dysregulated cancer cells metabolism constitutes a nearly universal feature of many types of cancer cells including PCa. Many tumors exhibit deficiencies in one or more amino acid synthesis or salvage pathways forcing a reliance on the extracellular pool of these amino acids to satisfy their metabolic demands. Cysteine is sulfur containing non-essential amino acid and previous studies highlighted that depletion of this amino acids led to the elevated intracellular levels of ROS in cancer cells [14]. To counterbalance high levels of ROS, tumor cells enhance the production of ROS scavenging GSH [16]. Cysteine is one of the building blocks of GSH, elevated production of GSH may exhaust endogenous sources of Cystine [17]. Subsequently, to survive and proliferate, cancer cells accelerate the uptake of extracellular cystine (disulfide forms of cysteine) via the CSSC/Glu antiporter (xCT transporter) [17, 18]. This research perspective highlights those depleting amino acids in cancer cells likely enhances treatment response. Our therapeutic strategy approaches highlight four mechanistic views.

**Figure 1:**
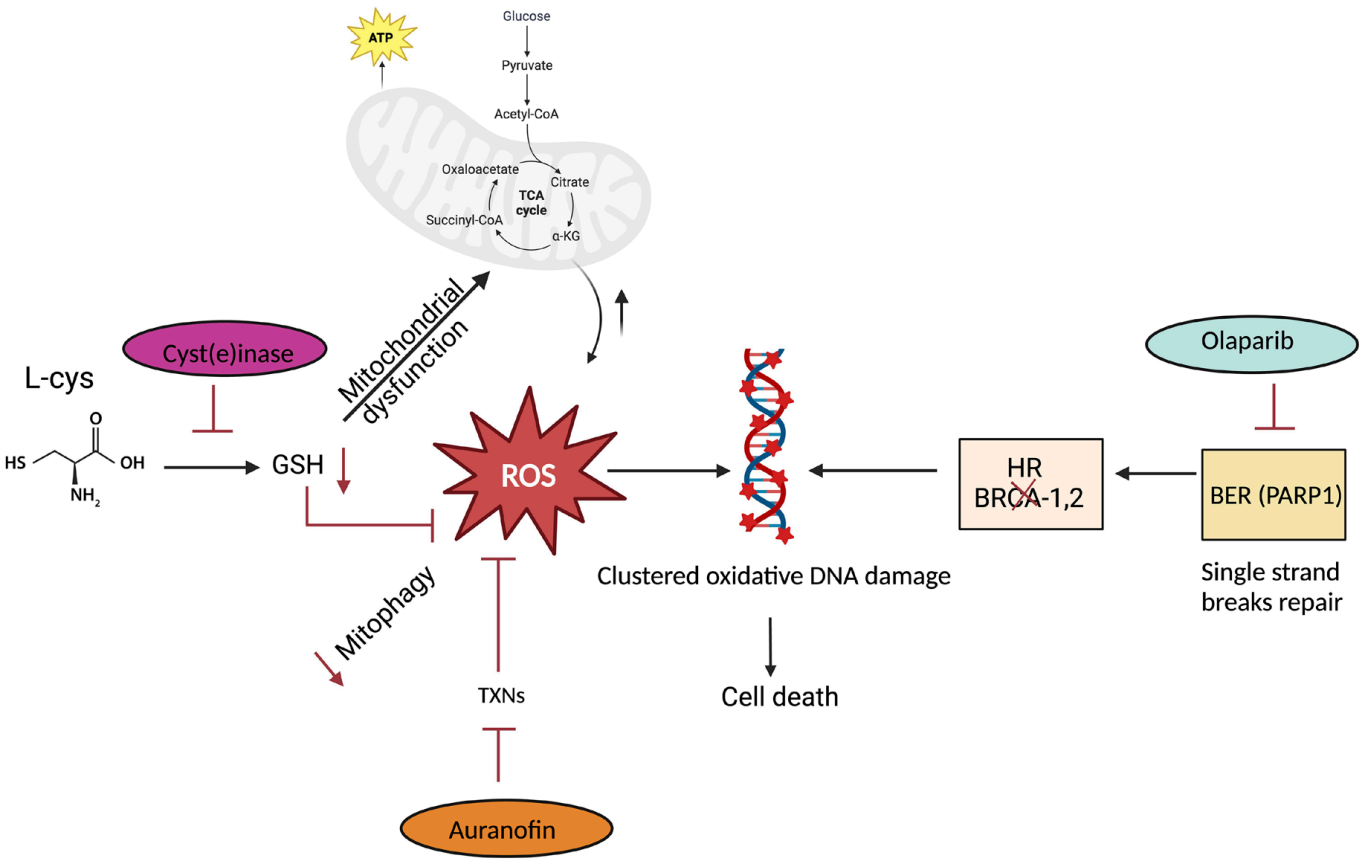
Amino acid depletion induce DNA damage and enhance sensitivity of the cancer cells to DNA repair and/or antioxidant inhibitor (created with https://www.biorender.com/). Cyst(e)inase mediated depletion of cysteine/cystine leads to downregulation of antioxidant GSH. This leads to mitochondrial dysfunction and oxidative stress. However, cells overcome the mitochondrial ROS accumulation by utilizing alternative antioxidants defense mechanism i.e., thioredoxin reductase. Therefore, the first strategy is to target thioredoxin reductase by auranofin increases the oxidative stress and mitophagy. The second strategy is inhibiting, one of the BER factor, PARP1 to block the ROS-induced ssDNA breaks repair and generate dsDNA breaks. The third approach is exploiting the DNA repair deficiency of the cancer cells such as BRCA defective cancer cells. In this scenario, Cyst(e)inase treatment alone or in combination with either TXNs inhibitors or PARP1 inhibitor sensitizes the PCa cells.

**Figure 2:**
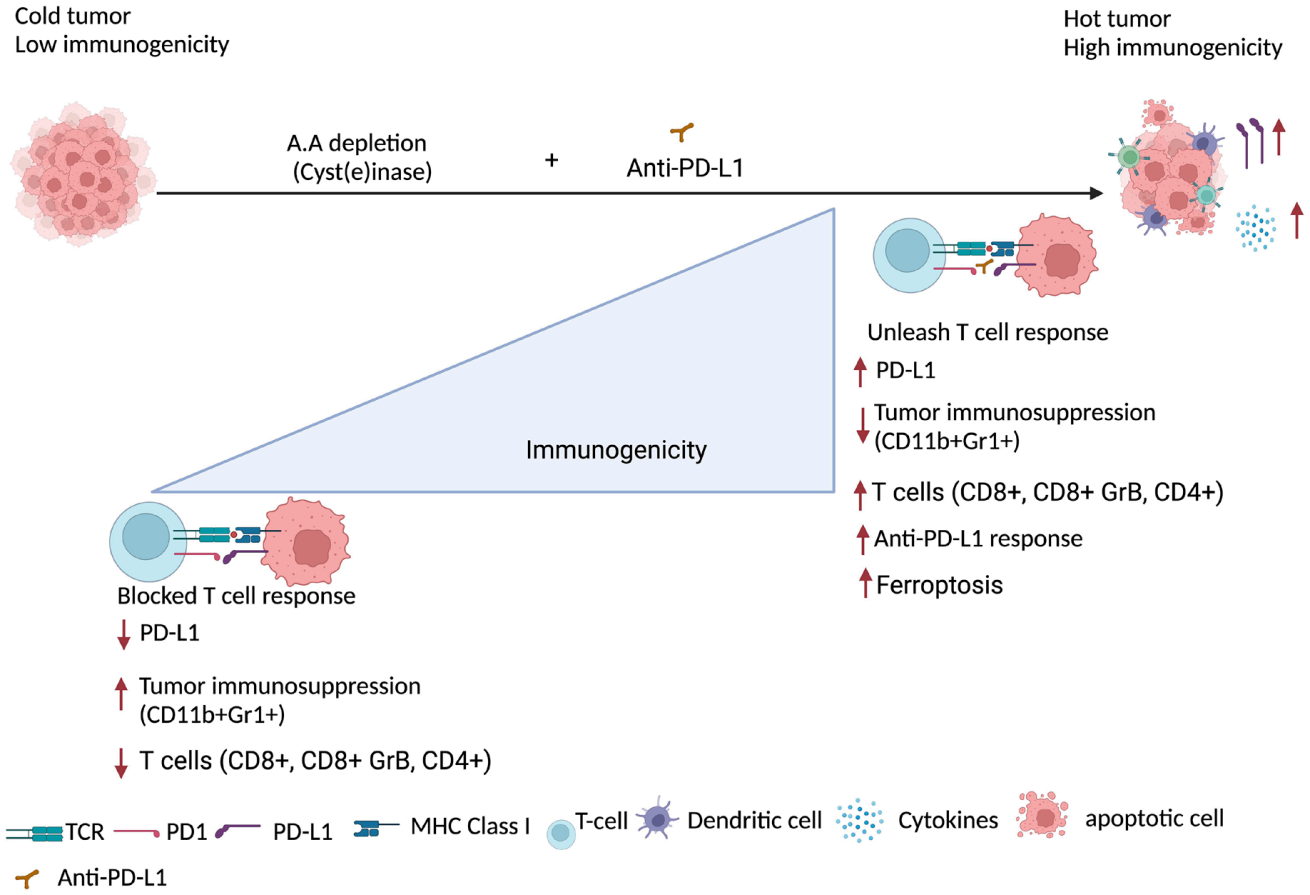
Amino acid starvation enhances the efficacy of immunotherapy (created with https://www.biorender.com/). Limiting Cysteine transportation in cancer cells by Cyst(e)inase results in reduced cellular GSH that causes clustered oxidative DNA damage [34]. In addition to DNA damage, it increases the infiltration of immune cells including the CD8+ T cells, CD8+Gr8 + cytotoxic T cell as well as triggers the ferroptosis of tumor cells in tumor microenvironment [61]. Thus, combining the amino acid depletion strategy with anti-PD-L1 therapy have synergistic effect and can convert immunologically cold tumors to hot tumors.

(i) *Would it possible to push the cancer cells over the edge by increasing oxidative stress and targeting antioxidant defense to the level that they cannot recover?* Cellular redox potential is largely determined by glutathione (GSH), which accounts for more than 90% of cellular thiols [19]. GSH is primarily found in the cytosol except for a small percentage in the mitochondria [20] and is the primary antioxidant responsible for maintaining the intracellular microenvironment essential for normal cellular function including scavenging ROS. As noted above, cancer cells are known to have high levels of ROS, such as superoxide anion that can lead to oxidative DNA damage and that can be detrimental to cancer cell survival [21]. Thus, cancer cells must maintain adequate levels of antioxidant defenses (e.g., GSH levels) for growth and survival. In support of this thesis, several studies have achieved selective killing of transformed cells through perturbation of redox status [22–24]. However, the upregulation of antioxidant capacity in cancer cells can confer drug resistance. It is well document that GSH protect the cancer cells against oxidative injury by reducing H_2_O_2_ and scavenging ROS [25]. Notably, mitochondrial GSH depletion induces increased mitochondrial ROS exposure which impairs bioenergetics and leads to cell death [26–28]. Several inhibitors of cyst(e)ine transporter including an FDA approved drug sulfasalazine have shown [26] efficacy on GSH depletion [29]. Moreover, combination treatments of sulfasalazine and vitamin C has demonstrated decrease in GSH and an increase in ROS level in prostate cancer [30]. While oxidative stress plays a dominant role in GSH depletion in cancer cells, some are causally related to reduced expression of GSH synthetic enzymes [13]. Low concentration of ROS act as signaling molecules to activate proliferation and survival pathways [31]. If the level of ROS reaches above a certain threshold level that may exert a genotoxic and cytotoxic effect, leading to the death of cancer cells and thus limiting cancer progression [32, 33]. Therefore, reducing GSH levels could be a promising approach to increase the oxidative stress within cancer cells, potentially enhancing the effectiveness of cancer treatment (Figure 1). Treating the cancer cells with Cyst(e)inase enhances ROS accumulation, decrease GSH level and alter the cell cycle progression pattern [14, 34]. Furthermore, combinatorial treatment of the cells with the agent that target antioxidant proteins such as Thioredoxins (TXNs) that scavenge ROS by cycling between oxidized and reduced forms with the help of TXNRs [35, 36] provide alternative therapeutic alternative. It is evident that mammals have two major isoforms, a cytosolic TXN1 and a mitochondrial TXN2, that pair up with TXNR1 and TXNR2, respectively [37]. Auranofin, which is an FDA approved drug (Ridaura^®^) for the treatment of rheumatoid arthritis that inhibits both isoforms of TXNR [38]. In our recent work, we target ROS scavenging molecules such as TXNR GHS with Auranofin in combination of with Cyst(e)inase synergistically enhance the accumulation of ROS and reduced PCa cell survival. (ii) *Is the ROS induced* via *amino acid depletion enough to induces DNA damage and inhibit DNA repair to synergize cancer cell death?* ROS are normal byproducts of numerous cellular processes, such as mitochondria metabolism [39]. At moderately increased levels, ROS induces DNA damage and promote genomic instability in cancer cells [40]. Recently, we reported that the growth of multiple cancer types *in vivo*, including PCa, is inhibited by increasing ROS through administration of an engineered human enzyme, Cyst(e)inase, that degrades extracellular cysteine (L-Cys) and cystine (CSSC) and subsequently decreases the intracellular levels of L-Cys and GSH [14, 41]. Cyst(e)inase induced ROS causes clustered oxidative DNA damage in cancer cells (Figure 1), which is similar to other previous studies [42]. Our biochemical characterization of depleting cysteine/cystine provides the opportunity to enhances ROS associated clustered oxidative DNA damage that result in DSBs [34]. On the other hand, DNA repair mechanisms protect the cancer cells against ROS induced oxidative DNA damage or DNA replication stress [43, 44]. Our data suggested that repair of DNA lesions induced by ROS requires the interaction of different DNA repair pathways, including base excision repair (BER) [45]. BER is the predominant pathway for repair of ROS-induced DNA base damage such as 8-oxo-dG [46]. Clustered oxidative DNA damage can also occur (i.e., oxidized DNA bases in proximity) and when processed by BER can lead to DSBs [47]. We have found that because of elevated intracellular ROS levels, Cyst(e)inase-treated PCa cells accumulate both single strand and double strand DNA breaks (SSBs and DSBs, respectively) due to increased oxidative DNA damage. The presence of both SSBs and DSBs in PCa cells treated with Cyst(e)inase suggests that in addition to BER, other DNA repair pathways may also likely play a role in protecting cells from this damage. Based on the PCa genomic profiling DNA repair targeted therapies including poly-ADP ribose polymerase (PARP) inhibitors now FDA-approved for the treatment of select men with metastatic castration resistant prostate cancer (CRPC) harboring specific aberrations in DNA repair genes [48]. In our study, we have shown that combination of Cyst(e)inase with Olaparib result in accumulation of DNA damage and reduces the survival of the PCa cells [34] (Figure 1). (iii) *Can we harness genetic vulnerability of the cancer cell to increase sensitivity to amino acid depletion?* Molecular-targeted therapies and treatment stratification using genetic landscape of cancer cells have rapidly gained momentum in cancer therapy. A significant proportion of prostate cancers harbor DNA damage repair (DDR) and DNA damage response deficiency. DNA damage repair (DDR) pathways are commonly impaired in prostate cancer [49] with a prevalence of germline mutations among men with metastatic PCa reported to be ~12% [50]. In addition, analysis of The Cancer Genome Atlas (TCGA) reveals that 19% of primary prostate cancers have mutations in DNA repair genes [51]. Other genome sequencing studies conducted on metastatic PCa tissue samples have shown that 23% had defects in DNA repair genes (i.e., *BRCA1/2, ATM, CDK12, FANCA, FANC, PALB2, ATR, RAD51B*, and *RAD51C*) [52]. For example, the recent genomic analysis by Robinson et al. [52] has revealed that mutations in *BRCA2* which is involved in the HR DNA repair pathway are observed in 13.3% of primary prostate tumors. Our study has shown that Cyst(e)inase treatment in HR deficient (BRCA2−/−) prostate cancer accumulated double strand breaks and resulted in a significant decrease of cancer cell survival [34]. This part of the study unraveled how cancer cells genetic liability enhances amino acid depletion-based treatment response. (iv) *Can we apply combinatorial treatment approaches in amino acids depleted cancer cells to enhance immunotherapy response?* PCa is recognized as a poorly immunogenic tissue with immunological cold with low antigen-presenting process and T-cell activation and recruitment of immunosuppressive cells [53]. The development of immunotherapy in cancer treatment has brought an exciting era of anti-prostate cancer therapy through antitumor immune responses. Immune checkpoint blockade (ICB) has shown limited benefit in prostate cancer in several studies [54, 55]. Nonetheless, durable objective responses have been reported, suggesting that patients with molecularly defined subsets of prostate cancer may benefit from this therapeutic approach [56]. Pembrolizumab, an antibody targeting the programmed cell death protein 1 (PD-1) receptor, recently earned accelerated approval by FDA for the treatment of microsatellite instability–high (MSI-H) or mismatch repair deficient (dMMR) solid tumors, independent of site of origin [57]. Notably, prostate tumors with HR gene mutations confers sensitivity to PARP inhibitors [52, 58, 59] and correlates with increased immune checkpoint expression [60]. Wang et al. demonstrated that Cyst(e)inase treatment in combination with ICB, enhances anti-tumor immunity by elevating the infiltration of CD8+ and CD4+ cells, along with promoting tumor ferroptosis [61]. Shah A, et al. shows that Cyst(e)inase treatment increase the expression of PD-L1 in prostate tumor (Figure 2). Furthermore, combinatorial treatment of PCa with Cyst(e)inase in combination with ant-PD-L1 antibody shows that increase infiltration of cytotoxic T cell and reduce tumor size *in vivo* model [34]. This discovery may open opportunity to explore the unknown outcomes via pharmacological depletion of amino acids in cancer cells to benefit from combinatorial therapeutic approaches. Overall identifying the metabolic need or manipulating the amino acid need of the cancer cells likely generate a better opportunity to enhance the tumor immunogenicity and increase the efficacy of the ICB response.

### Future works

Dietary restriction is typically not sufficient to achieve a therapeutically relevant level of amino acid depletion for cancer treatment. Therefore, pharmacological intervention using enzymes to deplete non-essential amino acids in tumor microenvironment for therapeutic purposes provides alternative therapeutic potential. Depleting amino acid likely expose the Achilles heel of the cancer and exacerbate DNA repair targeted and immune based therapy response. Furthermore, the genetic vulnerability of the tumor provides a better platform to enhance sensitivity to amino acid depletion-based treatment. Integrative analyses combining genomics with other features such as metabolic demand to exploit the tumor microenvironment, could be used for therapeutic opportunity to benefit from current therapeutic models and inform the development of novel therapeutic biomarker-driven therapy approaches for cancer patients. In the future, exploring additional non-essential amino acid demand of tumor decoding and engineering other molecular profiling may help to fulfill unmet needs for predictive biomarkers in novel immunotherapeutic approaches.
